# Development of a New Index to Assessing the Safety Culture Based on the Edgar Schein Model (Audit Approach)

**DOI:** 10.1155/tswj/3166187

**Published:** 2025-09-24

**Authors:** Davood Eskandari, Abbas Haghparast Ghomesheh, Mousa Jabbari, Abdollah Gholami, Omran Ahmadi

**Affiliations:** ^1^Department of Occupational Health and Safety, School of Health, Shahrekord University of Medical Sciences, Shahrekord, Iran; ^2^Department of Occupational Health and Safety, School of Public Health and Safety, Shahid Beheshti University of Medical Sciences, Tehran, Iran; ^3^Department of Occupational Health and Safety, School of Health, Birjand University of Medical Sciences, Birjand, Iran; ^4^Department of Occupational Health and Safety, Faculty of Medical Science, Tarbiat Modares University, Tehran, Iran

**Keywords:** audit approach, safety culture, spherical FAHP

## Abstract

**Introduction:** Over the past few decades, particularly following the Chernobyl incident, awareness of the importance of safety culture has increased significantly. Traditionally, safety culture has been assessed using questionnaires, with results often influenced by employees' psychological states. Therefore, the present study evaluates safety culture through an audit-based approach, employing performance indicators grounded in Edgar Schein's model.

**Materials and Methods:** To identify the key factors contributing to the safety culture index, Edgar Schein's model was applied. Relevant indicators were derived across three levels: artifacts, espoused values, and basic assumptions. The fuzzy analytic hierarchy process (FAHP), combined with expert evaluations, was used to determine the relative importance of these criteria. Finally, the company's safety culture index was quantitatively assessed using guide tables and the weighted contribution of each factor.

**Results:** Then, 10 indicators were identified across the three levels of Schein's model: quality of documentation and procedures, employee involvement, management of professional competence, change management, organizational learning, openness and communication, prioritization of safety, managerial knowledge, safety perspectives, and attitudes toward mistakes. According to the FAHP results, prioritization of safety had the highest weight (0.157), while quality of documentation and procedures had the lowest weight (0.026) in the safety culture index.

**Discussion:** An audit-based approach proves more effective than questionnaire methods for quantitatively assessing safety culture.

## 1. Introduction

Safety culture has emerged as one of the most important advancements in occupational safety and health research in recent years. It is closely associated with safety-related behaviors in workplaces such as manufacturing, shipping, chemical processing, and building maintenance [1–4]. Safety culture is also a significant predictor of occupational accident and injury rates in the oil and gas industry [4, 5]. While safety culture is not the sole determinant of safety within an organization, it plays a fundamental role in promoting safe behaviors among workers [6]. In other words, an organization's collective understanding and response to safety issues are more effective than relying on individual decision-making alone. This is because safety culture reflects the shared beliefs, values, and learned practices that are embedded in both the conscious and unconscious minds of employees [7].

Improving the organization's safety culture leads to better proactive safety management, reducing the need for the organization to wait for errors to occur [8]. A positive safety culture encourages open discussion of issues and improvement strategies involving all employees and does not tolerate deviations from operational procedures [9].

Safety culture is a crucial element of organizational life and should be treated like other important aspects and measured. The assessment of safety culture can be conducted using either subjective or objective methods [10–12]. Subjective assessments may include questions about behavioral safety norms, error reporting, assignment of resources, and the management's commitment to safety [13, 14].

Some questionnaires that claim to assess safety culture include dimensions and statements closely resembling those used to measure safety climate. However, self-reported questionnaires are unlikely to provide an accurate measure of safety culture; instead, they often reflect respondents' current emotional state or feelings about the workplace [15, 16]. The concept of safety culture is inherently complex, lacking a single straightforward indicator to capture its status. The multilevel nature of culture, combined with the ambiguous characteristics of some cultural layers, further complicates its measurement.

Using indices to measure safety culture offers several potential advantages, including the ability to track temporal trends and increased managerial focus on the aspects being measured. Despite the inherent challenges and limitations associated with measuring safety culture, it remains advisable to identify and apply appropriate indicators [17].

Edgar Schein's model of safety culture provides a valuable framework for understanding and addressing the complexities of organizational safety behavior by illustrating how cultural levels span from the readily observable to the implicit and unseen.

### 1.1. Edgar Schein's Model of Safety Culture

**Figure 1:**
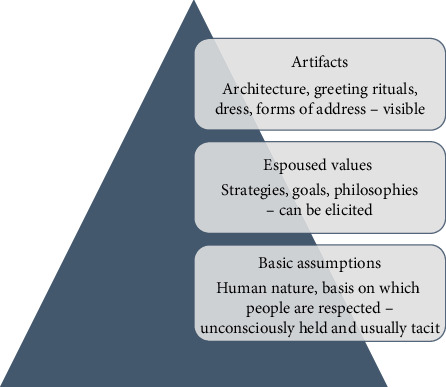
Three levels of culture.

Edgar Schein categorizes organizational culture into three levels: artifacts, espoused values, and basic assumptions (Figure 1). In this framework, artifacts represent the most visible and accessible aspects of culture. These include what one sees, hears, and feels when entering an organization, such as its architecture and design. At this level, culture is tangible and can evoke an immediate emotional response. However, it is often unclear why an organization is structured in a particular way or why individuals behave as they do. Understanding these deeper dynamics requires moving beyond artifacts to the next level of culture.

Espoused values refer to the stated principles and beliefs that an organization or its members explicitly endorse and support. These values can be identified through observation or inquiry. They reflect the ideals that individuals claim to uphold. Common examples within organizations include commitments to equal opportunity, teamwork, employee empowerment, and prioritizing safety.

At the deepest level, basic assumptions form the foundation of culture. These are deeply ingrained, often unconscious beliefs that are widely shared within a cultural group. To truly understand an organization's culture, it is essential to uncover these underlying assumptions. While influenced in part by national culture, basic assumptions are also shaped by the organization's history, as well as the values, beliefs, and assumptions of its founders and key leaders who have contributed to its development and success.

The present study is aimed at developing a set of objective safety culture indicators to assist Iranian companies in assessing their safety culture without relying on employee perception surveys. The key subindices are organized according to the levels defined by Edgar Schein's safety culture model. Data will be collected through an audit-based approach, and the final safety culture index (SCI) will be calculated to provide a quantitative evaluation of the organization's safety culture, minimizing dependence on subjective judgments.

## 2. Materials and Methods

In this study, several steps were undertaken to develop an index for evaluating an organization's safety culture.

### 2.1. Step 1: Identifying Influencing Factors

The International Atomic Energy Agency (IAEA) provides guidance on establishing and maintaining safety culture in nuclear facilities, as outlined in IAEA-TECDOC-1329. This document defines the main categories that serve as key indicators in the present study. These indicators are organized into three groups—artifacts, espoused values, and basic assumptions—following Edgar Schein's model of organizational culture.

This framework was informed by research findings, lessons learned from organizational failures in safety management, and international collaboration among safety experts (Table 1). Given the study's audit-based methodology and the availability of supporting evidence and documentation, the research team selected indicators that could be reliably assessed using existing evidence.

### 2.2. Step 2: Scoring the Identified Factors

Detailed instructions were developed to define each main indicator and its associated characteristics or criteria, referred to as subindicators. Each subindicator was assessed using a 5-point Likert scale, ranging from the ideal to the weakest state, with scoring guides provided in tables to support the audit process.

The ideal state was carefully described to ensure consistent, reliable assessments and to minimize subjective interpretation by auditors. This description was informed by multiple sources, including CCPS and OSHA guidelines on safety management system documentation, performance indicators, and audit practices. Additional references included the ISO 45001 standard and materials from the websites and documentation of leading global companies such as Shell and British Petroleum.

To define the full scale, the ideal state was first established, after which the most systematic characteristics were sequentially removed or downgraded to delineate progressively weaker states.

### 2.3. Step 3: Weighing of the Influencing Factors

A fuzzy hierarchical analysis approach was employed to determine the relative weights of the factors contributing to the SCI, recognizing that not all factors carry equal importance. The analytic hierarchy process (AHP) is a well-established method for multicriteria decision-making (MCDM) that allows complex problems to be structured hierarchically while considering both quantitative and qualitative criteria in the evaluation process.

Fuzzy set theory is often integrated into MCDM models to address the inherent ambiguity and uncertainty in decision-making. In this study, we applied the spherical fuzzy analytic hierarchy process (SF-AHP) to calculate the weights of the identified factors within the developed SCI.

The proposed approach consists of five key steps:

1. Clearly defining the problem and establishing the hierarchical structure.
2. Constructing a spherical fuzzy pairwise comparison matrix.

The spherical fuzzy pairwise ratios (SFPRs) were computed by comparing each element at a given level to elements at the higher level using the circular fuzzy preference scale presented in Table 2. For each criterion and subcriterion, pairwise comparisons were performed. Decision-makers used the linguistic scale in Table 2 to assess the relative importance of each criterion.

3. Checking the consistency.

The pairwise comparison matrix composed of spherical fuzzy values is transformed into a pairwise comparison matrix consisting of crisp positive reciprocal points, using the formula *R* = *ρi* *j*, where *ρi* *j* = *μi* *j*/*υi* *j*.

To ensure the reliability of the comparisons, consistency of the resulting matrix is assessed using Saaty's eigenvalue method.

4. Prioritized the criteria

The following steps were used to weigh the criteria and alternatives for each comparison matrix:

i. The aggregation processes of SFSs compute the spherical fuzzy geometric mean for the row (Equation 1):

 $$\begin{array}{c} r^{i}={\left(\overset{n}{\underset{j=1}{\prod }}r_{ij}\right)}^{1/n} \end{array}$$

i. Find the weight of each aggregated row *ℱ* (*r*^*i*^) was used Equation (2), and prioritization function, $ℱ\;\left(\tilde{A_{s}}\right)=\mu \left(1-\nu \right)\left(1-\pi \right).$ Then, the total weight was computed:
ii. ∑_*i*=1_^*n*^*ℱ*(*r*^*i*^) calculating the priority vector W = [*𝓌*_1_, *𝓌*_2_, ⋯, *𝓌*_*n*_], was by means of equation 3:

 $$\begin{array}{c} w_{i}=F\left(r^{i}\right)/\overset{n}{\underset{i=1}{\sum }}F\left(r^{i}\right) \end{array}$$

### 2.4. Step 5: Determining Total Priorities and Ranking

The final scores for each alternative were calculated using the formula: *W*_*i*_ = ∑*𝓌*_*j*_ *𝓌*_*ij*_, where *W*_*i*_ is the utility of the *i* the alternative, *𝓌*_*j*_ is the weight of the *j* the criterion, and *𝓌*_*ij*_ is the performance score of the *i* the alternative for the *j* the criterion.

Based on these final scores, the alternatives were ranked from highest to lowest priority.

### 2.5. Step 4: Case Study

A new framework was developed based on the identification of key factors and their corresponding scoring system to evaluate safety culture. To demonstrate its practical application, this framework was implemented in a detergent manufacturing company located in Tehran. The company was selected as a representative example of the chemical manufacturing sector, allowing the audit-based approach to be tested in a real-world industrial setting.

Through this case study, the safety culture of the organization was systematically assessed, and the specific conditions and actions required to achieve the highest possible score for each indicator were identified and outlined. The results provide practical insights for similar companies seeking to strengthen their safety culture using objective, evidence-based methods.

## 3. Results

According to Edgar Schein's model, safety culture indicators are classified into three levels: artifacts, espoused values, and basic assumptions. The research team selected these indicators based on their relevance and the availability of evidence and documentation suitable for audit purposes.

At the artifact level, several factors were identified, including the quality of documentation and procedures, employee involvement, management of professional competence, and management of change. The selected espoused values comprised organizational learning, openness and communication, and placing a high priority on safety.

For basic assumptions, the focus was on managers' knowledge, views on safety, and perceptions of mistakes.  Table 3 presents the key indicators categorized according to the different levels of safety culture.

**Figure 2:**
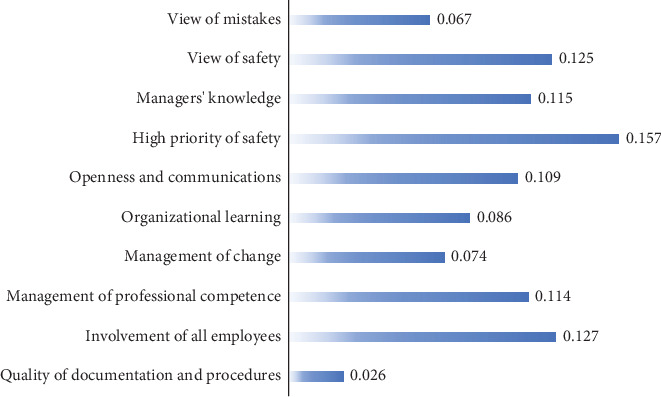
Weighting factors of criteria affecting the safety culture.

The safety culture indicators were evaluated by 16 experts, including university faculty members, safety managers from reputable companies, and industry professionals with over 10 years of experience, through pairwise comparisons. According to the results, the highest weighted factors influencing safety culture were identified as follows: high priority of safety (weight = 0.157), involvement of all employees (weight = 0.127), and view of safety (weight = 0.125) (Figure 2). Additionally, the consistency index was calculated as 0.01, which is less than the acceptable threshold of 0.1, indicating a satisfactory level of consistency in the evaluations.

By quantifying the relative importance, or weights, of the various safety culture indicators, it becomes possible to calculate a comprehensive and objective SCI for the organization. This index provides a numerical representation of the overall safety culture performance, integrating the contribution of each indicator according to its weighted significance. The final SCI can be computed using the following equation, which aggregates the weighted scores of all evaluated factors.

 $$\begin{array}{c} Safety\;culture\;index=\left(0.157*x_{1}\right)+\left(0.127*x_{2}\right)+\left(0.125*x_{3}\right)+\left(0.115*x_{4}\right)+\left(0.114*x_{5}\right)+\left(0.109*x_{6}\right)+\left(0.086*x_{7}\right)+\left(0.074*x_{8}\right)+\left(0.067*x_{9}\right)+\left(0.026*x_{10}\right), \end{array}$$

where *x*_1_–*x*_10_ represents the score for high priority of safety, the involvement of employees, the view of safety, managers' knowledge, management of professional competency, openness and communication, organizational learning, management of change, view of mistakes, and quality of documentation and procedures.

Each of these criteria is rated on a Likert scale ranging from 1 to 5, and the overall SCI falls within the same range.

Following the determination of the relative importance of these safety culture indicators, an audit was conducted on the 10 identified indicators within the studied company. The audit assessed the score of each indicator across the three levels of Schein's model, as summarized in Table 4. After assessing the importance of safety culture indicators, an audit was conducted on the 10 identified indicators in the understudy company. The audit determined the score of each of these indicators in three different levels of Schein's model (Table 4).

To calculate the final score of the SCI, the average scores obtained for each subcriterion were multiplied by their respective weighting factors. This weighting reflects the varying degrees of influence that each factor exerts on the overall safety culture of the organization. By applying these weighted values, the SCI provides a comprehensive and nuanced quantitative measure that accounts for both the performance levels and relative importance of all evaluated safety culture components. This approach ensures that more critical factors have a proportionally greater impact on the final index, allowing for a more accurate and meaningful assessment of the organization's safety culture.

$$\begin{array}{cc} \; & \text{SCI}=\sum \left(\text{weighting load}\times \text{factor scores}\right),\\ \text{SCI}=\left(0.157*3.50\right)+\left(0.127*2.33\right)+\left(0.125*4.27\right)+\left(0.115*2.66\right)+\left(0.114*3.80\right)+\left(0.109*4.28\right)+\left(0.086*3.83\right)+\left(0.074*4.83\right)+\left(0.067*3.57\right)+\left(0.026*4.12\right),\\ \text{SCI}=3.62. \end{array}$$

Based on the audit results, the calculated SCI for the studied organization was 3.62. According to the predefined criteria, all indicator scores are expected to be equal to or greater than 3 to be considered within an acceptable range. However, the scores for two critical factors—managers' knowledge and involvement of all employees—fell below this threshold, indicating areas of concern.

As a result, the overall SCI does not meet the desired target range of 4–5, which signifies a strong and mature safety culture. This finding highlights specific weaknesses within the organization's safety management system, particularly in leadership engagement and workforce participation. Addressing these gaps is essential for improving the organization's safety culture and achieving higher levels of safety performance.

Future efforts should focus on enhancing managerial training and increasing employee involvement initiatives to foster a more positive and effective safety culture. These targeted improvements can help raise the SCI to the desired levels and contribute to a safer work environment.

## 4. Discussion

There are various qualitative and quantitative tools available for measuring safety culture, with questionnaires being the most commonly utilized methodology [18–20]. Comparing this study with others reveals that many researchers across different case studies have primarily relied on questionnaires and employees' opinions to assess the safety culture within companies. Notable examples include studies by Parkestani et al. [21], Raisi et al. [22], Fazli et al. [23], Hosseinzadeh et al. [24], Nouri et al. [25], Mohammadfam et al. [26], Sepehr et al. [27], and Khoshakhlagh et al. [28].

For example, Mohammadfam et al. [29] investigated the effects of technical and managerial interventions on safety culture by comparing conditions before and after these interventions. Similarly, other studies such as those conducted by Fleming et al. [30], Tappura et al. [31], Rowen et al. [32], Sherratt et al. [33], and Gong et al. [34] gathered expert opinions on safety culture through questionnaire-based approaches.

Despite the widespread use of questionnaires in safety culture research, the present study adopts an audit-based approach grounded in Edgar Schein's model to determine the weights of safety culture indicators. The limitations of questionnaire methods have been noted in the literature, where they are often deemed ineffective in fully revealing the true essence of an organization's safety culture [35, 36]. This is apparent in the factors identified by questionnaires, which frequently reflect a general assessment of management practices but fall short in providing deeper insights into the underlying cultural assumptions that shape organizational behavior [35, 37].

It is important to distinguish safety climate from safety culture. Safety climate can be understood as a temporary snapshot of the organization's atmosphere and is primarily reflected in employees' perceptions. In contrast, safety culture represents deeper, more enduring values and assumptions that underpin organizational practices [18]. According to Schein [38], the core of an organization's culture lies at its foundational level and must be interpreted through various sources, including but not limited to the organizational climate [38].

Given the complexity of safety culture, measuring it with a single simple indicator is not feasible. The multilevel nature of culture, combined with the implicit and tacit characteristics of some layers—especially basic assumptions—makes its measurement challenging. However, using carefully selected indicators to measure safety culture offers significant advantages. Such indicators enable the identification of trends over time and tend to increase managerial attention and commitment to the aspects being measured. Consequently, this fosters greater organizational engagement with the concept of safety culture. As safety culture is a fundamental aspect of organizational life, it deserves to be treated with the same rigor and measurement practices as other critical organizational components.

## 5. Conclusion

This research focused on prioritizing and weighting key safety culture indicators, followed by conducting an audit within an organization to assess its safety culture. The findings offer a robust and practical framework that other organizations can adopt to systematically evaluate their own safety culture levels. By utilizing this criterion, organizations are equipped with an objective tool to identify strengths and areas for improvement in their safety culture.

To build upon this study, it is recommended that similar safety culture assessments using the audit-based approach grounded in Edgar Schein's three-level model be conducted across diverse industries and organizational contexts. Such studies would help validate the applicability and effectiveness of this method in different settings. Moreover, exploring alternative MCDM techniques—such as the AHP and the technique for order of preference by similarity to ideal solution (TOPSIS)—and comparing their outcomes with those of the present study could provide valuable insights into the relative advantages and limitations of these methodologies. This comparative analysis would further enhance the development of reliable, comprehensive tools for safety culture evaluation.

## Figures and Tables

**Table 1:** Characteristics of safety culture.

Artifact value level	Top management commitment to safety; visible leadership; systematic approach to safety; self-assessment; strategic business importance of safety; absence of safety versus production conflict; relationship to regulators and other external groups; proactive and long term perspective; management of change; quality of documentation and procedures; compliance with regulations and procedures; sufficient and competent staff; employees have a questioning attitude; man, technology and organization knowledge; clear roles, responsibilities and accountabilities; motivation and job satisfaction; involvement of all employees; good working conditions with regard to time pressure, workload and stress; measurement of safety performance, proper resource allocation; collaboration and teamwork; handling of conflict; relationship between managers and employees; awareness of work process; good housekeeping.
Espoused value level	High priority to safety; safety can always be improved; openness and communications; organizational learning
Basic assumption level	Time focus; view of mistakes; view of safety; systems thinking; role of managers; view of people.

**Table 2:** The spherical fuzzy terms and illustration.

**Linguistic term**	**(** ***μ***, **υ**, ***π*)**	**Geometrical representation**
Certainly high	(0.9, 0.1, 0.1)	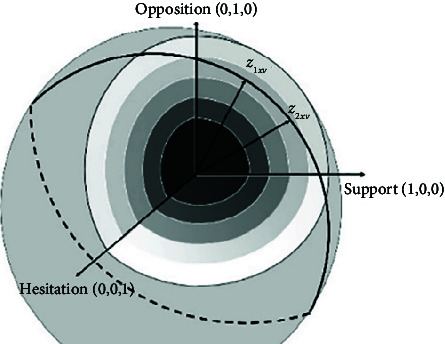
Very high	(0.8, 0.2, 0.2)	
High	(0.7, 0.3, 0.3)	
Above average	(0.6, 0.4, 0.4)	
Average	(0.5, 0.5, 0.5)	
Below average	(0.4, 0.6, 0.4)	
Low	(0.3, 0.7, 0.3)	
Very low	(0.2, 0.8, 0.2)	
Certainly low	(0.1, 0.9, 0.1)	

**Table 3:** Safety culture levels.

Artifacts	Quality of documentation and procedures	Documentation should be easy to understand and comprehensive. Responsibilities for creating and reviewing documentation should be clearly defined. Documentation should be utilized for both training and work purposes.
	Involvement of employees	Employees will not feel responsible for safety if they are not included in identifying and solving safety issues. The organization should have procedures in place to involve employees in safety-related issues.
	Management of professional competence	All persons working for an organization must be competent to perform their assigned OH&S roles. OH&S training needs shall be identified, and steps taken to enhance competence, where any gaps are identified.
	Management of change	Being aware of the challenges related with organizational change is crucial. The presence of an organized process for implementing change will be substantial evidence that this matter is being dealt with.

Espoused values	Organizational learning	The organization consistently reassesses the environment and adjusts in anticipation of environmental changes. Commitment to organizational learning is vital for understanding safety issues and uncovering their root causes.
	Openness and communications	Good communication is essential within an organization for effective employee performance. Employees must feel trusted with knowledge and have the opportunity to express concerns, either as a group or individually.
	High priority of safety	Many organizations claim safety as their top priority, but their actions do not always confirm it in practice.

Basic assumptions	Managers' knowledge	Managers should possess adequate safety knowledge to confidently address safety issues with employees, and the organizational hierarchy should not hinder any manager from openly supporting safety enhancements.
	View of safety	The responsibility for safety falls on every employee and does not rest solely with managers or the regulator.
	View of mistakes	Mistakes can be seen as opportunities for learning or for punishment. It is crucial for safety that employees feel comfortable pointing out safety mistakes without fear of punishment.

**Table 4:** Safety culture scores.

**Indicators**	**Scores**
*Quality of documentation and procedures*	
Operational areas have adequate and documented implementation procedures in place.	4
The procedures and instructions are kept up-to-date and revised as needed.	4
Communicating revisions in procedures and instructions to users.	5
Providing clear communication of safety procedures and safety instructions.	4
Comprehensibility of safety procedures and instructions for users.	4
Applying personnel knowledge in creating and revising safety regulations and instructions.	2
Integration of safety procedures with operational procedures.	5
Development and implementation of the work permit system.	5
*Involvement of employees*	
Participation of nonmanagement level employees in key meetings such as safety committee and risk assessment meetings.	3
Reviewing the recommendations put forward by the employees to enhance workplace safety.	3
Feedback to employees on the results of safety audits, measurements and risk assessments.	1
*Management of professional competence*	
Existence of a system to identify the competency characteristics of employees.	3
Setting clear goals for training programs.	5
Providing adequate training in technical fields, safety subjects including human factors and the nature of safety and accidents, and uncertainties and risks.	5
Holding a sufficient number of supplementary courses on safety and technical issues.	5
Adequate ways to introduce new personnel.	3
Ensuring the appropriateness of the scope, content, and quality of educational programs.	2
Receive feedback from trainees and use it in developing the training program.	4
Using simulators and simulated operations in education.	3
Reviewing the events of the company and outside the company in the form of learning lessons of the accidents.	4
The existence of a suitable recruitment method to identify professional qualification needs.	4
*Management of change*	
A clear definition of a technical change or an organizational change.	5
Considering the rate and speed of changes when planning changes.	5
Conducting a risk assessment for organizational changes before implementing that change.	5
Anticipate issues related to usability and maintainability of new technology, tools, and modifications during the design and implementation phases	5
Considering human and organizational factors in change management.	5
Ensuring organizational memory is not destroyed by changes (such as documents).	4
*Organizational Learning*	
Existence of a comprehensive system for reporting incidents and other learning experiences such as pseudo-accidents.	5
Having a systematic corrective action plan to deal with deviations.	5
Collecting and analyzing the operating experience of other similar companies.	1
Existence of methods to identify new vulnerabilities.	5
Having a system to collect personnel development initiatives.	2
Existence of a system to investigate and analyze internal incidents, taking into account technical, human, and organizational factors.	5
*Openness and communications*	
Providing feedback to personnel about accidents and near-accidents.	5
Disseminate adequate information on safety issues received from other organizations.	5
Reminding personnel about safety issues in meetings and internal communications.	5
Regularly informing the personnel about the situation and safety challenges.	5
Having open communication about positive and negative issues in the organization.	5
Existence of formal and informal communication channels to increase safety concerns in the organization	2
Communicating the importance of various safety rules and procedures clearly to the personnel.	3
*High priority of safety*	
Senior management visits the workplace regularly.	1
Conducting sufficient inspections of safety issues to prevent workplace accidents.	5
Clearly considering the safety of employees of high importance by the manager.	5
Considering safety as important as production (sales) by management.	3
*Knowledge of managers*	
Regularly receiving leadership training and safety promotion according to the individual needs of managers.	2
Holding general and specific safety training courses to improve the knowledge level of managers.	1
Training middle managers to improve basic safety skills.	5
*View of safety*	
Creating uncompromising expectations to achieve excellent safety performance.	4
Integration of leading and lagging indicators to monitor the organization's safety performance.	5
Rewarding important achievements in the field of safety.	5
Requirement to develop annual safety management action plans.	5
Using safety performance as a key criterion for increasing salaries, bonuses, and promotions.	5
Measurement, force rating, and public display of peer safety performance.	4
Annual organization of 360° safety surveys of managers and supervisors.	3
Begin meetings by discussing current safety performance status and areas of concern.	5
Regular integration of safety messages into written correspondence.	4
The need for immediate 24-h verbal notification of serious incidents.	5
Ensuring adequate staffing for performance (EHS).	2
*View of mistakes*	
How to plan correction after an error by the manager.	3
Comprehensive analysis of errors.	5
Using the results of error analysis to improve the work process.	3
Referring to colleagues when people alone are not able to correct an error.	3
Sharing people's mistakes with others by the organization so that they do not make mistakes.	3
Continuation of the work of employees who admit their mistakes without problems.	3
The existence of a framework to prevent errors in the organization.	5

## Data Availability

The data used to support the findings of this study can be obtained from the corresponding author upon reasonable request.
